# Comprehensive B-Cell Immune Repertoire Analysis of Anti-NMDAR Encephalitis and Anti-LGI1 Encephalitis

**DOI:** 10.3389/fimmu.2021.717598

**Published:** 2021-10-07

**Authors:** Jingjing Feng, Siyuan Fan, Yinwei Sun, Haitao Ren, Hongzhi Guan, Jing Wang

**Affiliations:** ^1^ CAS Key Laboratory of Mental Health, Institute of Psychology, Chinese Academy of Sciences, Beijing, China; ^2^ Department of Psychology, University of Chinese Academy of Sciences, Beijing, China; ^3^ Department of Neurology, Peking Union Medical College Hospital, Chinese Academy of Medical Sciences and Peking Union Medical College, Beijing, China

**Keywords:** anti-N-methyl-D-aspartate receptor encephalitis, anti-leucine-rich glioma-inactivated 1 encephalitis, B-cell immune repertoire, common clones, adaptive immune responses

## Abstract

Anti-N-methyl-D-aspartate receptor encephalitis (anti-NMDARE) and anti-leucine-rich glioma-inactivated 1 encephalitis (anti-LGI1E) are the two most common types of antibody-mediated autoimmune encephalitis. We performed a comprehensive analysis of the B-cell immune repertoire in patients with anti-NMDARE (*n* = 7) and anti-LGI1E (*n* = 10) and healthy controls (*n* = 4). The results revealed the presence of many common clones between patients with these two types of autoimmune encephalitis, which were mostly class-switched. Additionally, many differences were found among the anti-NMDARE, anti-LGI1E, and healthy control groups, including the diversity of the B-cell immune repertoire and gene usage preference. These findings suggest that the same adaptive immune responses occur in patients with anti-NMDARE and anti-LGI1E, which deserves further exploration.

## Introduction

Anti-N-methyl-D-aspartate receptor encephalitis (anti-NMDARE) is mediated by antibodies against NMDA receptors. The main binding site of anti-NMDAR IgG is the N368/G369 amino acids at the N-terminal of the NR1 subunit of NMDA receptors (1–3). The main classes of pathogenic antibodies in anti-NMDARE are IgG1 and IgG3, which are synthesized intrathecally (4). The positive rate of anti-NMDAR IgG in the cerebrospinal fluid of patients is nearly 100% (4), but this rate is only 71.4%–85.6% in serum samples (5, 6). There are two main triggers of anti-NMDARE: tumor, usually a teratoma of the ovary, and herpes simplex virus infection (7–9); approximately 50% of young women with this disease have an ovarian teratoma (10). There is also evidence indicating that NR1 antigens are expressed in anti-NMDARE patients with a history of teratoma (11, 12). Additionally, approximately 50% of the patients have unknown immunologic triggers (8). Previous studies have revealed that anti-NMDAR antibodies can alter the normal interaction between NMDA receptors and EphB2, displacing these receptors from synaptic to extrasynaptic sites, resulting in internalization of NMDA receptors (8, 13). The internalization of NMDA receptors leads to a reduction in NMDA receptor-mediated synaptic currents, impaired long-term potentiation, and a syndrome characterized by encephalopathy, memory deficits, and other neuropsychiatric manifestations (8, 13). The typical clinical symptoms of patients with anti-NMDARE include rapidly progressive psychiatric symptoms, cognitive impairment, seizures, abnormal movements, or coma (14). Approximately 80% of patients with anti-NMDARE recover or substantially improve with immunotherapy directed to remove the antibodies and antibody-producing plasma cells (corticosteroids, intravenous immunoglobulins, plasma exchange, rituximab, or cyclophosphamide), tumor resection (when needed), and symptomatic care (13).

Anti-leucine-rich glioma inactivated 1 encephalitis (anti-LGI1E) is mediated by antibodies against LGI1, a component of the voltage-gated potassium channel complex (1). Antibodies in patients with anti-LGI1E mostly recognize the leucine-rich repeat or the epitempin repeat domains of LGI1 (15). Therefore, epitopes of anti-LGI1 encephalitis are relatively more complex and diverse. Previous studies have revealed that the main pathogenic role of anti-LGI1 antibodies is to inhibit ligand–receptor interactions between LGI1 and ADAM22 or ADAM23, decreasing the total and synaptic levels of the voltage-gated potassium channels Kv1.1 and alpha-amino-3-hydroxy-5-methyl-4-isoxazole propionate receptors (AMPAR), resulting in a severe impairment of neuronal transmission, plasticity, and memory (13, 16, 17). The main subclass of antibodies is IgG4 (16), and the sensitivity of antibody detection in serum is higher than that in cerebrospinal fluid (100% vs. 88%) (14, 18). Moreover, 5%–10% of patients with anti-LGI1E have thymoma (1). Seizures and cognitive disturbances are common initial symptoms of anti-LGI1E, and faciobrachial dystonic seizures are typical of anti-LGI1E (16). Immunotherapy for anti-LGI1E is similar to that for anti-NMDARE, and approximately 70% of patients had a favorable outcome at ≥2 years of follow-up (16).

Immune repertoire studies have been used to identify potential diagnostic markers and help understand the pathogenesis of diseases (19–21). Few studies have examined the B-cell immune repertoire in patients with anti-NMDARE and anti-LGI1E (15, 22–24). These studies have revealed the pathogenicity of anti-NMDAR antibodies and anti-LGI1 antibodies and provided evidence suggesting that the anti-LGI1 antibodies are synthesized intrathecally. Little is known about how the B-cell repertoire changes in patients with these two types of autoimmune encephalitis. To identify potential biomarkers for anti-NMDARE and anti-LGI1E and help understand their pathogenesis with respect to the immune repertoire, we performed a comparative analysis of peripheral circulating B cells in patients with these two types of autoimmune encephalitis and healthy individuals.

## Materials and Methods

### Study Design

Peripheral blood samples from 7 patients with anti-NMDARE (1 patient provided blood samples from the first episode and at recurrence: N12→N22, and 1 patient provided blood samples from before and after the first immune treatment: N26→N32) and from 10 patients with anti-LGI1E were collected from December 2019 to November 2020. Peripheral blood samples from four healthy controls (HC) were also collected. This study was approved by the Institutional Review Board of Peking Union Medical College Hospital (IRB JS-891). Written informed consent was obtained from each patient or their legal surrogate. All patients with anti-NMDARE and anti-LGI1E fulfilled the diagnostic criteria of definite autoimmune encephalitis proposed by Graus et al. (1). The cerebrospinal fluid and serum samples of patients were tested for anti-NMDAR IgG and anti-LGI1E IgG antibodies by indirect immunofluorescence using EU 90 cells transfected with the NR1 subunit of the NMDAR complex or LGI1 and immobilized on commercially available biochips (EUROIMMUN, Lübeck, Germany). The clinical information of patients is summarized in **Table 1**.

**Table 1:** Clinical features of studied subjects.

Category	Anti-NMDARE (*n* = 7)	Anti-LGI1E (*n* = 10)	HC (*n* = 4)
Age (years)	30.43 ± 3.4	55.1 ± 9.07	36.3 ± 4.8
Female:male	3:4 (42.86%:57.14%)	3:7 (30%:70%)	2:2 (50%:50%)
Abnormal mental behavior	7 (100%)	0	0
Conscious disturbance	4 (57.14%)	0	0
Seizure	6 (85.71%)	10 (100%)	0
Memory deficit	4 (57.14%)	6 (60%)	0
Tumor	0	0	0
Psychiatric symptoms	0	1 (10%)	0
FBDS	0	3 (30%)	0
IVIg	6 (85.71%)	8 (80%)	0
Steroids	6 (85.71%)	8 (80%)	0
MMF	1 (14.29%)	4 (40%)	0
Antibody positivity in cerebrospinal fluid	7 (100%)	3 (30%)	0
Antibody positivity in serum	2 (28.57%)	9 (90%)	0

Anti-NMDARE, anti-N-methyl-D-aspartate receptor; anti-LGI1E, anti-leucine-rich glioma-inactivated 1; HC, healthy control; FBDS, faciobrachial dystonic seizures; IVIg, intravenous immunogloblin; MMF, mycophenolate mofetil.

### Flow Cytometry

All fresh samples were transported at 4°C. Peripheral blood samples were mixed with an equal volume of 0.9% NaCl and centrifuged at 800*g* for 20 min using a Lymphoprep separation solution (Axis-Shield, Norway) to obtain peripheral blood mononuclear cells. The next steps were performed using previously published protocols (25). Briefly, B cells were labeled with various antibodies [NR1-FITC, OriGene; anti-CD19-percp/Cy5.5, Biolegend; LGI1-green fluorescent protein (GFP), our lab] and 4′,6-diamidino-2-phenylindole for sorting and counting. FlowJo software (version 10.7) was used for quantitative analysis of the flow cytometry, and the numbers of NR1-positive and LGI-positive B cells were calculated in 100,000 peripheral blood mononuclear cells from each sample.

### LGI1-GFP Recombinant Protein Preparation

The recombined plasmid pcmv3-lgi1-gfp (SinoBiological) was generated with ClonExpress (Vazyme), and “linker” and “His” tag sequences were added after the GFP sequence to obtain the complete expression sequence of LGI1-GFP-His. The recombinant plasmid was transfected (Vazyme) into HEK 293T cells, and the supernatant from the culture medium after cell lysis was collected 72 h later. LGI1-GFP-His recombinant protein was purified with Ni-NTA resin (TransGen).

### Single B-Cell Receptor Sequencing

Sequences of the B-cell receptor (BCR) heavy chains of NR1-positive and LGI1-positive B cells were obtained according to a previously reported method (25). Briefly, reverse transcription of single B cells was performed. Then, two rounds of nested polymerase chain reaction were conducted for BCR amplification. The products of polymerase chain reaction were gel extracted, ligated, and cloned, and the sequences were obtained by Sanger sequencing.

### High-Throughput Bulk Sequencing of the BCR Heavy Chain

Bulk sequencing of the BCR heavy chain was performed by CapitalBio Technology *via* the following steps: cDNA was obtained by reverse transcription with OligodT with 1 μg of mRNA from peripheral blood samples (for samples with less than 1 μg of mRNA, a volume of 10 μl was used to prepare libraries); second-strand synthesis was performed with IGH constant domain-specific primers and FR3 region-specific primers for BCR heavy chains (not shown). Illumina adapters were added by polymerase chain reaction amplification. The library was quantified and purified by Qubit 3.0 and gel extraction, and finally, raw data were obtained by 150-base paired-end Illumina sequencing. More than 6,000,000 reads were sequenced for each sample, and the number of times each clone was recovered was positively correlated with its degree of clonal expansion.

### Processing of Raw Reads

Raw data were analyzed using MiXCR (26) to identify productive clones. VDJtools (27) (version 3.0.13) was used to analyze the gene usage preference of IGHV, IGHJ, and the IGHV–IGHJ gene combination. The proportions of reads of each IGHV gene, IGHJ gene, and IGHV–IGHJ gene combination were calculated and compared among the anti-NMDARE, anti-LGI1E, and HC groups. The somatic hypermutation rate of the BCR (FR3 region) was calculated by the *Defineclone.py* of the Change-O toolkit (28). The amino acid length of complementary determining region 3 (CDR3) was analyzed and plotted using VDJtools and the R package (version 4.0.2) *immunarch*.

The Gini coefficient is commonly used to measure income inequality in economic studies, and it has also been used to measure the equality of distribution in individual immune repertoires (29, 30). A Gini coefficient of 0 indicates equal frequencies of all clones, and a Gini coefficient of 1 indicates a monoclonal sample. The Gini coefficient of each isotype was calculated using the R package *immunarch* and was used to compare the degree of clonal expansion in the repertoire of each sample (30).

Chao1 is a non-parametric asymptotic estimator of species richness (number of species in a population) (31). The normalized Chao1 estimate calculated using VDJtools was used to assess the diversity of the immune repertoire. Normalization was performed by downsampling datasets to the size of the smallest dataset and computing the estimate for the resulting dataset. Analysis of clonality showed the proportions of hyperexpanded clones and rare clones. Chao1 and clonality were analyzed using the R package *immunarch*.

Similarities of the B-cell immune repertoire (common clones, similar clones, Morisita index, *t*-distributed stochastic neighbor embedding clustering) were analyzed and plotted using the R package *immunarch*. Common clones were defined if the sequences were encoded by the same BCR IGHV/IGHJ gene segments and possessed identical amino acids in the CDR3 region. Sequences that possessed identical amino acids in the CDR3 region were defined as similar clones.

### Statistical Analysis

Statistical analysis was performed using R (version 4.0.2). The Kruskal–Wallis test was used for multiple group comparisons, and Holm–Bonferroni correction was performed to determine the adjusted *P*-value (*P*_adj_). A *P*_adj_-value <0.05 was considered statistically significant. Comparisons of numbers of antigen-positive B cells between the HC and anti-NMDARE groups and between the HC and anti-LGI1E groups were performed using the Mann–Whitney test. Moreover, a value of *P <*0.05 was considered statistically significant.

## Results

### Autoimmune Encephalitis-Associated BCR Clones

**Figure 1:**
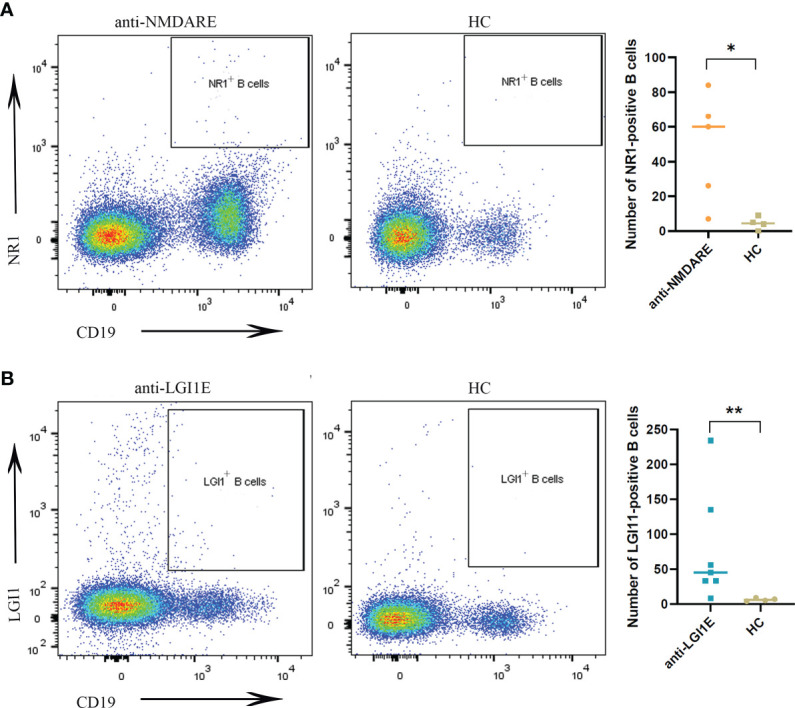
Comparison of NR1-positive B cells and LGI1-positive B cells in peripheral blood samples among the anti-NMDARE, anti-LGI1E, and HC groups. **(A)** Results of flow cytometric analysis of NR1-positive B cells in the anti-NMDARE and HC groups. **(B)** Results of flow cytometric analysis of LGI1-positive B cells in the anti-LGI1E and HC groups. Anti-NMDARE, anti-N-methyl-D-aspartate receptor; anti-LGI1E, anti-leucine-rich glioma-inactivated 1; HC, healthy control. *P < 0.05, **P < 0.01.

The results of flow cytometric analysis showed significantly higher NR1-positive B-cell (CD19^+^NR1^+^) levels in the peripheral blood of patients with anti-NMDARE than those of the HC group (*P* = 0.0317) (**Figure 1A**), and the level of LGI1-positive B cells (CD19^+^LGI1^+^) in the peripheral blood of patients with anti-LGI1E was also significantly higher than that of the HC group (*P* = 0.0091) (**Figure 1B**). Some partial BCR clones of antigen-positive B cells were identified in patients by single-cell sequencing, and the sequences are summarized in **Supplementary Table 1**.

Next, we analyzed the high-throughput data and did not find any BCR clones that were significantly overexpressed in the two encephalitis groups, but we found a few BCR clones with increased expression in the HC group (**Supplementary Table 2**).

### Basic Analysis of the B-Cell Immune Repertoire in Peripheral Blood Samples From the Anti-NMDARE, Anti-LGI1E, and HC Groups

The basic characteristics of the sequencing data of the BCR heavy chain, including the number of total reads and total unique BCR clones, from patients with anti-NMDARE and anti-LGI1E and HC are summarized in **Table 2**. On average, 21,665,199, 12,431,273, and 17,668,993 reads were sequenced from the anti-NMDARE, anti-LGI1E, and HC groups.

**Table 2:** Summary of BCR heavy chain sequencing data from the anti-NMDARE, anti-LGI1E, and HC groups.

Group	Subject	Number of total reads	Number of unique BCR clones
HC	H1	19,443,564	477,441
	H2	17,097,390	511,128
	H3	18,088,592	89,273
	H4	16,046,426	837,891
Anti-LGI1E	L1	12,871,142	81,116
	L13	18,662,269	26,088
	L15	11,821,081	64,711
	L18	17,404,636	316,828
	L2	7,239,595	25,883
	L24	11,022,181	38,722
	L3	10,936,758	21,600
	L5	11,105,016	15,721
	L6	9,813,657	4,541
	L7	13,436,392	6,478
Anti-NMDARE	N12	25,491,885	37,532
	N14	30,465,831	79,895
	N22	23,508,911	208,781
	N23	26,085,766	294,683
	N26	20,844,353	220,347
	N32	13,418,562	5,624
	N33	24,117,896	89,593
	N37	20,513,964	36,504
	N41	10,539,620	66,187

Anti-NMDARE, anti-N-methyl-D-aspartate receptor; anti-LGI1E, anti-leucine-rich glioma-inactivated 1; HC, healthy control.

**Figure 2:**
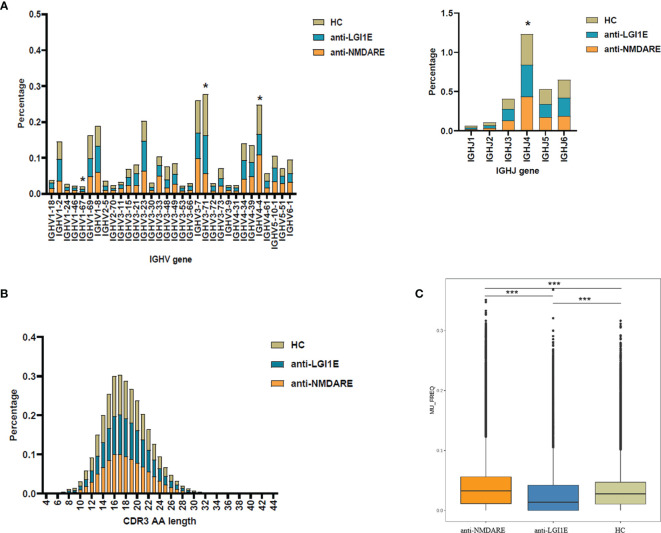
Basic analysis of the B-cell immune repertoire in peripheral blood samples from the anti-NMDARE, anti-LGI1E, and HC groups. **(A)** Analysis of IGHV and IGHJ gene usage preferences. **(B)** Analysis of the CDR3 amino acids length. **(C)** Analysis of the somatic hypermutation rate. Anti-NMDARE, anti-N-methyl-D-aspartate receptor encephalitis; anti-LGI1E, anti-leucine-rich glioma-inactivated 1 encephalitis; HC, healthy control; AA, amino acids. *P_adj_ < 0.05, ***P_adj_ < 0.001.

Analysis of the characteristics of IGHV and IGHJ gene usage preference (**Figure 2A** and **Supplementary Table 3**) showed that patients with anti-NMDARE had significant differences compared with HC, including increased IGHJ4 (*P*_adj_ = 0.028) and decreased IGHV3-71 (*P*_adj_ = 0.036) levels; furthermore, patients with anti-NMDARE had significant differences compared with patients with anti-LGI1E, including increased IGHV1-67 (*P*_adj_ = 0.043) and IGHV4-4 (*P*_adj_ = 0.006) levels.

After comparing the usage frequency, significant differences in a total of 5 IGHV–IGHJ gene combinations were observed between patients with anti-NMDARE and HC (**Table 3**). Among them, IGHV1-3_IGHJ1 was used more frequently in patients with anti-NMDARE, whereas the other combinations (four IGHV–IGHJ gene combinations, namely, IGHV3-48_IGHJ1, IGHV3-49_IGHJ1, IGHV3-48_IGHJ3, IGHV5-78_IGHJ3) exhibited an increased usage frequency in HC. Additionally, significant differences in 15 IGHV–IGHJ gene combinations were observed between patients with anti-LGI1E and HC (**Table 3**), whereas 14 IGHV–IGHJ gene combinations exhibited significantly higher frequencies in HC, namely IGHV3-64_IGHJ1, IGHV4-39_IGHJ1, IGHV7-4-1_IGHJ1, IGHV3-43_IGHJ2, IGHV3-64_IGHJ2, IGHV3-48_IGHJ3, IGHV3-64_IGHJ3, IGHV3-64_IGHJ4, IGHV3-73_IGHJ4, IGHV3-49_IGHJ5, IGHV3-64_IGHJ5, IGHV3-71_IGHJ5, IGHV5-78_IGHJ5, and IGHV3-64_IGHJ6, and only IGHV1-2_IGHJ5 was used more frequently in patients with anti-LGI1E. Additionally, 14 IGHV–IGHJ gene combinations with significant differences were observed between patients with anti-NMDARE and patients with anti-LGI1E; most of these combinations were used more frequently in patients with anti-NMDARE, including IGHV6-1_IGHJ1, IGHV1-69D_IGHJ2, GHV4-4_IGHJ2, IGHV3-73_IGHJ3, IGHV4-4_IGHJ3, IGHV1-67_IGHJ4, IGHV3-11_IGHJ4, IGHV3-7_IGHJ4, IGHV5-51_IGHJ4, and IGHV1-67_IGHJ5.

**Table 3:** Summary of IGHV–IGHJ gene combinations with significant difference among the anti-NMDARE, anti-LGI1E, and HC groups.

IGHV–IGHJ	*P*-value (**Kruskal–Wallis test**)	*P*_adj_ (pairwise comparison)	Note
IGHV1-3_IGHJ1	0.032*	0.027*	Anti-NMDARE > HC
IGHV3-48_IGHJ1	0.044*	0.039*	HC > anti-NMDARE
IGHV3-49_IGHJ1	0.037*	0.031*	HC > anti-NMDARE
IGHV3-64_IGHJ1	0.036*	0.032*	HC > anti-LGI1E
IGHV4-39_IGHJ1	0.041*	0.035*	HC > anti-LGI1E
IGHV6-1_IGHJ1	0.028*	0.035*	Anti-NMDARE > anti-LGI1E
IGHV7-4-1_IGHJ1	0.013*	0.010*	HC > anti-LGI1E
IGHV1-69D_IGHJ2	0.025*	0.038*	Anti-NMDARE > anti-LGI1E
IGHV3-43_IGHJ2	0.005**	0.009*	HC > anti-LGI1E
IGHV3-64_IGHJ2	0.036*	0.032*	HC > anti-LGI1E
IGHV4-4_IGHJ2	0.011*	0.008**	Anti-NMDARE > anti-LGI1E
IGHV3-48_IGHJ3	0.008**	0.028*	HC > anti-NMDARE
		0.009**	HC > anti-LGI1E
IGHV3-64_IGHJ3	0.036*	0.032*	HC > anti-LGI1E
IGHV3-73_IGHJ3	0.027*	0.023*	Anti-NMDARE > anti-LGI1E
IGHV4-4_IGHJ3	0.009**	0.008*	Anti-NMDARE > anti-LGI1E
IGHV5-78_IGHJ3	0.031*	0.028*	HC > anti-NMDARE
IGHV1-67_IGHJ4	0.013*	0.013*	Anti-NMDARE > anti-LGI1E
IGHV3-11_IGHJ4	0.023*	0.026*	Anti-NMDARE > anti-LGI1E
IGHV3-64_IGHJ4	0.036*	0.032*	HC > anti-LGI1E
IGHV3-7_IGHJ4	0.045*	0.040*	Anti-NMDARE > anti-LGI1E
IGHV3-73_IGHJ4	0.011*	0.011*	HC > anti-LGI1E
IGHV4-4_IGHJ4	0.006**	0.004**	Anti-NMDARE > anti-LGI1E
IGHV5-51_IGHJ4	0.031*	0.029*	Anti-NMDARE > anti-LGI1E
IGHV1-2_IGHJ5	0.034*	0.035*	Anti-LGI1E > HC
IGHV1-67_IGHJ5	0.010*	0.020*	Anti-NMDARE > anti-LGI1E
IGHV3-49_IGHJ5	0.020*	0.037*	HC > anti-LGI1E
IGHV3-64_IGHJ5	0.036*	0.032*	HC > anti-LGI1E
IGHV3-71_IGHJ5	0.034*	0.040*	HC > anti-LGI1E
IGHV5-78_IGHJ5	0.029*	0.035*	HC > anti-LGI1E
IGHV6-1_IGHJ5	0.008**	0.015*	Anti-NMDARE > anti-LGI1E
IGHV2-26_IGHJ6	0.015*	0.025*	Anti-LGI1E > anti-NMDARE
IGHV2-5_IGHJ6	0.015*	0.025*	Anti-LGI1E > anti-NMDARE
IGHV3-64_IGHJ6	0.036*	0.011*	HC > anti-LGI1E

Anti-NMDARE, anti-N-methyl-D-aspartate receptor; anti-LGI1E, anti-leucine-rich glioma-inactivated 1; HC, healthy control.

*P_adj_ < 0.05, **P_adj_ < 0.01.

Analysis of the distribution of CDR3 amino acid lengths showed that the anti-NMDARE, anti-LGI1E, and HC groups had similar CDR3 amino acid distributions that approximated the Gaussian distribution (**Figure 2B**). The mean lengths in the HC, anti-NMDARE, and anti-LGI1E groups were 17.89, 18.20, and 17.99, respectively.

As shown in **Figure 2C**, a significant difference was observed in the somatic hypermutation rate of the FR3 region among the anti-NMDARE, anti-LGI1E, and HC groups (*P* = 2.2 × 10^−16^). The anti-NMDARE group had the highest somatic hypermutation rate (mean = 3.26 × 10^−2^), the anti-LGI1E group had a lower somatic hypermutation rate (mean = 1.39 × 10^−2^), and the HC group had a relatively high somatic hypermutation rate (mean = 2.78 × 10^−2^).

### Comparison of the Clonal Expansion per Isotype, Diversity, and Clonality of the B-Cell Immune Repertoire in Peripheral Blood Samples From the Anti-NMDARE, Anti-LGI1E, and HC Groups

**Figure 3:**
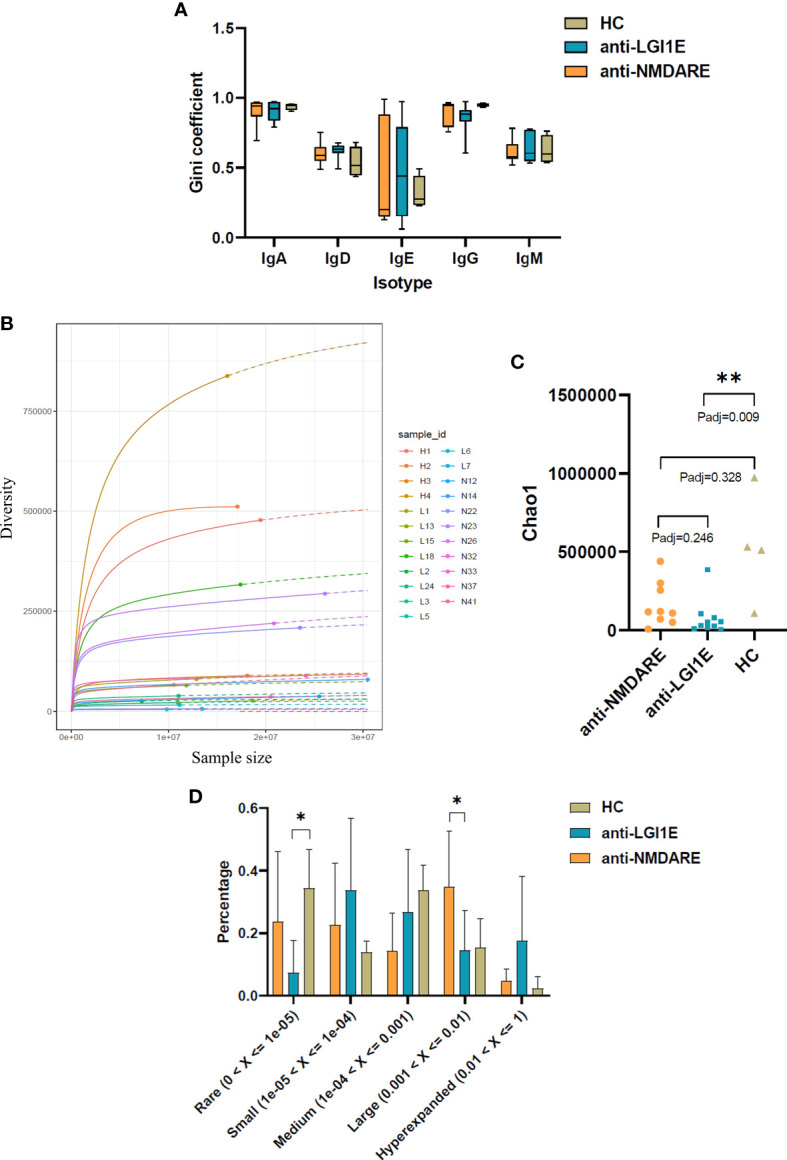
Comparison of the clonal expansion per isotype, diversity, and clonality of the B-cell immune repertoire in peripheral blood samples from the anti-NMDARE, anti-LGI1E, and HC groups using a multinomial model. **(A)** Gini coefficient of each isotype in the anti-NMDARE, anti-LGI1E, and HC groups. **(B)** Rarefaction analysis of BCRs showing the number of unique clones in a subsample plotted against its size (number of BCR cDNA molecules); solid and dashed lines indicate interpolated and extrapolated regions of rarefaction curves, respectively; points indicate the exact sample size and diversity. **(C)** Chao1 value of the anti-NMDARE, anti-LGI1E, and HC groups. **(D)** Analysis of clonality in each group. Anti-NMDARE, anti-N-methyl-D-aspartate receptor encephalitis; anti-LGI1E, anti-leucine-rich glioma-inactivated 1 encephalitis; HC, healthy control; BCR, B-cell receptor. *P_adj_ < 0.05, **P_adj_ < 0.01.

A comparison of the Gini coefficient per isotype showed no significant difference in clonal expansion of each isotype among the anti-NMDARE, anti-LGI1E, and HC groups (**Figure 3A**).

Rarefaction analysis of the repertoire showed that the degree of diversity in the HC group did not reach saturation, and the number of unique clones was significantly higher in the HC group than in the anti-LGI1E group (**Figure 3B**). A comparison of the diversity of repertoire using the normalized Chao1 estimate also showed that the HC group had the highest diversity (mean ± SEM = 530,865 ± 176,642) (**Figure 3C**), which was significantly higher than that in the anti-LGI1E group (mean ± SEM = 76,599 ± 35,949) (*P*_adj_ = 0.009). The HC group showed a trend toward greater diversity than the anti-NMDARE group (mean ± SEM = 163,558 ± 46,615), although the difference was not statistically significant (*P*_adj_ = 0.328).

The clonality of the immune repertoire is displayed in **Figure 3D** and **Supplementary Figure 2**. The HC group had more “rare clones” (0 < frequency < 1 × 10^−5^) than the anti-NMDARE and anti-LGI1E groups, especially compared with the anti-LGI1E group (*P*_adj_ = 0.040), and this was consistent with the result of Chao1 value. The anti-NMDARE group had more “large clones” (0.001 < frequency < 0.01) than the anti-LGI1E group (*P*_adj_ = 0.026), and the anti-LGI1E group seemed to have more “hyperexpanded clones” (0.01 < frequency < 1).

### Similarity of the B-Cell Immune Repertoire in Peripheral Blood Samples From the Anti-NMDARE, Anti-LGI1E, and HC Groups

**Figure 4:**
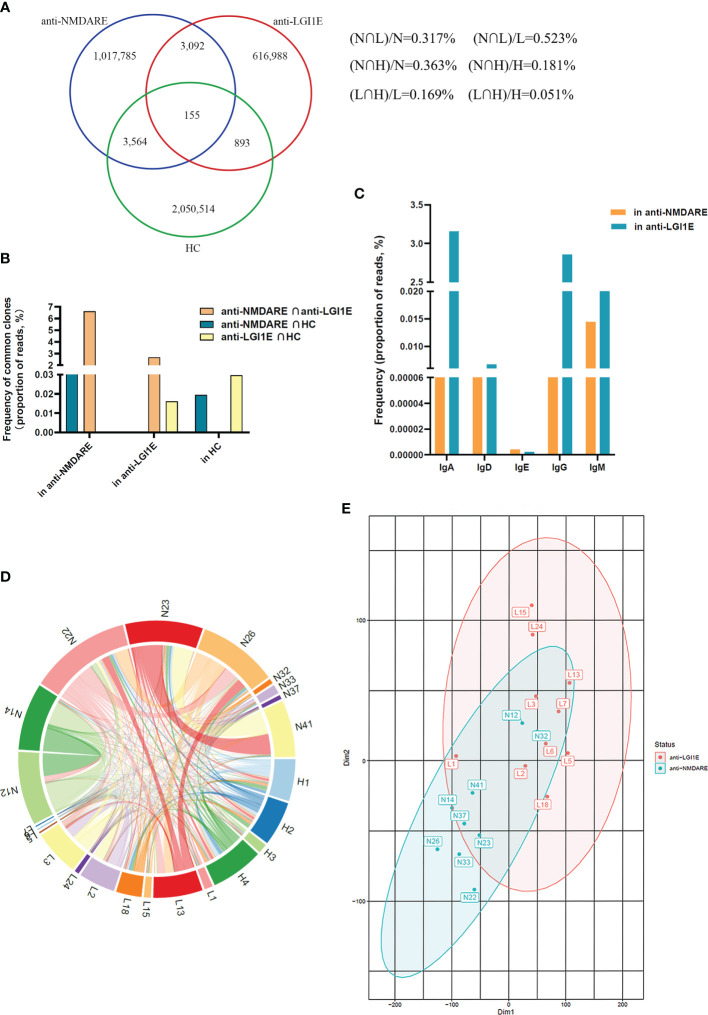
Comparison of the similarity of the B-cell immune repertoire in peripheral blood samples from the anti-NMDARE, anti-LGI1E, and HC groups. **(A)** Number of common clones among the anti-NMDARE, anti-LGI1E, and HC groups in a Venn diagram. **(B)** The proportions of reads of common clones shared by each two groups of the anti-NMDARE, anti-LGI1E, and HC groups. **(C)** The result of frequency of common clones shared by the anti-NMDARE and anti-LGI1E groups for different isotypes (IgA/IgD/IgE/IgG/IgM) through analyzing the proportion of reads. **(D)** Number of common clones in each sample from the anti-NMDARE, anti-LGI1E, and HC groups in a circos plot; the area of each color represents the number of unique clones shared by two samples. **(E)** *t*-stochastic neighbor embedding plot of the BCR in samples from the anti-NMDARE and anti-LGI1E groups. Anti-NMDARE, anti-N-methyl-D-aspartate receptor encephalitis; anti-LGI1E, anti-leucine-rich glioma-inactivated 1 encephalitis; HC, healthy control; BCR, B-cell receptor.

Overall, 1.02, 0.62, and 2.06 million unique BCR clones were identified in the anti-NMDARE, anti-LGI1E, and HC groups, respectively. We determined the degree of overlap between each group. There were 3,247 types of common clones shared by patients with anti-NMDARE and anti-LGI1E, and there were 3,719 types of common clones shared by patients with anti-NMDARE and HC (**Figure 4A**). Next, we calculated the proportions of reads of common clones shared by the anti-NMDARE, anti-LGI1E, and HC groups, and we found that the proportions of reads of common clones shared by these two encephalitis groups were the highest (**Figure 4B**). We calculated the frequency of these common clones shared by patients with anti-NMDARE and anti-LGI1E per isotype, and most of them were IgA/IgD/IgE/IgG (i.e., class-switched) (**Figure 4C**). To determine which samples contained these common clones, we analyzed the number of common clones in each sample (**Figure 4D**). Common clones shared by patients with anti-NMDARE and anti-LGI1E were not from a single sample, but rather from portions of both groups with varying degrees of clone overlap (**Figure 4D**). There were 7,317 types of common clones between N12 and N14 and 5,945 types of common clones between N23 and N41 (**Figure 4D**). There were 2,877, 2,087, 1,046, and 924 common clones between L13 and N26, L3 and N22, L18 and N23, and L2 and N22, respectively (**Figure 4D**). Additionally, N12 and N14 had the highest Morisita index of 7.84 × 10^−3^, and L2 had higher Morisita index of 4.41 × 10^−3^ and 6.14 × 10^−3^ with N12 and N14, separately (**Supplementary Figure 1**). It should be noted that N12 and N14 and the samples from the anti-LGI1E group were not in the same batch during library construction and sequencing. Additionally, we performed *t*-distributed stochastic neighbor embedding analysis of the BCR (**Figure 4E**), which indicated partial crossover between patients with anti-NMDARE and anti-LGI1E.

### Identification of Clones That Were the Same as or Similar to Common Clones Shared by Patients With Anti-NMDARE and Anti-LGI1E in Public BCR Datasets

Previous studies have shown that mining B-cell immune repertoire data can be used to identify highly similar antibodies with known functions (32–34). We next sought to determine whether these common clones shared by patients with anti-NMDARE and anti-LGI1E but not with HC contained biological signatures from past disease exposures. Therefore, we searched for these common clones in public databases containing BCR sequences.

**Figure 5:**
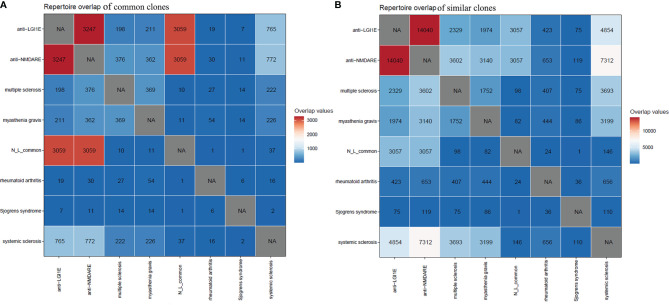
Common and similar clones among patients with anti-NMDARE, anti-LGI1E, myasthenia gravis, multiple sclerosis, rheumatoid arthritis, systemic sclerosis, and Sjögren’s syndrome. **(A)** The distribution of common clones in each group. **(B)** The distribution of similar clones in each group. Anti-NMDARE, anti-N-methyl-D-aspartate receptor encephalitis; anti-LGI1E, anti-leucine-rich glioma-inactivated 1 encephalitis; N_L_common, common clones shared by the anti-NMDARE and anti-LGI1E groups. NA, not applicable.

Initially, we searched for common clones of these two types of autoimmune encephalitis in GenBank (35) and found that some were similar to the sequences of clones in patients with myasthenia gravis. Therefore, we mined the BCR sequencing data of myasthenia gravis patients from the Sequence Read Archive database. Additionally, because anti-NMDARE and anti-LGI1E are autoimmune diseases, we examined BCR data from other autoimmune diseases from the Sequence Read Archive database. Public data from patients with myasthenia gravis, multiple sclerosis, rheumatoid arthritis, systemic sclerosis, and Sjögren’s syndrome were obtained. By analyzing common and similar clones among these autoimmune diseases, we found that few of these common clones shared by these two encephalitis groups appeared in the other autoimmune diseases (**Figure 5A**). However, there were a few common clones between patients with autoimmune encephalitis and systemic sclerosis (**Figure 5A**), and many similar clones were observed among the anti-NMDARE, anti-LGI1E, multiple sclerosis, myasthenia gravis, and systemic sclerosis groups (**Figure 5B**).

The Immune Epitope Database is a widely used resource for collecting BCR and TCR information (36). The database contains sequences of 1,825 types of BCRs and their antigens. We searched for common clones shared by patients with anti-NMDARE and anti-LGI1E in the Immune Epitope Database to identify antigens that can bind to these common clones, but none was found in this database.

## Discussion

We characterized the role of B cells in patients with anti-NMDARE and anti-LGI1E and HC at the BCR level. To our knowledge, this is the first study comparing the two most common types of autoimmune encephalitis by analyzing the B-cell immune repertoire. Identification of relatively high frequencies of common clones in BCR repertoires in patients with anti-NMDARE and anti-LGI1E was unexpected and interesting. Although there were also many common clones shared by the anti-NMDARE and HC groups, the proportion of reads of common clones shared by the anti-NMDARE and anti-LGI1E groups was the highest, indicating these clones had higher expansion in both of these two types of encephalitis. These two groups had relatively high BCR similarity (Morisita index and *t*-distributed stochastic neighbor embedding classification), and the majority of the common clones in these two types of encephalitis were class-switched, which may indicate that the same or similar adaptive immune responses occur in the circulating blood cells of patients with anti-NMDARE and anti-LGI1E. Although some of these common clones might be driven by exposure to common antigens (37), whether the same antigens were present in the anti-NMDARE and anti-LGI1E groups was not clear. Our study may be of importance for identifying similarities in the pathogenesis and treatment of these two types of autoimmune encephalitis.

Additionally, some common and similar clones were present in patients with anti-NMDARE, anti-LGI1E, systemic sclerosis, myasthenia gravis, and multiple sclerosis. Although the reason for the presence of common clones between these different types of autoimmune diseases is not known, our study has implications for future research on the existence of similar immune responses in different autoimmune diseases.

The results of flow cytometric analysis demonstrated that patients with anti-NMDARE or anti-LGI1E had more B cells in the peripheral blood that can combine with the NR1 antigen or LGI1 antigen than HC. Although the majority of patients with anti-NMDARE enrolled in this study had negative peripheral blood antibody tests, this does not mean that there were no NR1-positive B cells in their peripheral blood. Other than these antigen-positive BCR clones, we did not find other autoimmune encephalitis-related BCR clones by high-throughput sequencing as reported in previous studies (25, 38). This might result from the relatively small number of subjects included. Additionally, in the anti-NMDARE group, only two of the enrolled patients had serum antibody positivity. More patients with antibody-positive serum or cerebrospinal fluid samples may be needed.

Compared with the HC group, patients with anti-NMDARE or anti-LGI1E showed a decreased diversity of the B-cell repertoire, with the smallest value in the anti-LGI1E group. The two encephalitis groups also had lower Chao1 values, fewer rare clones, and more hyperexpanded clones, which suggests the existence of antigen-driven expanded BCR clones in autoimmune encephalitis. Other antigen-driven changes observed in these patients included the IGHV/IGHJ gene usage preference, IGHV–IGHJ combination usage preference, and somatic mutation rate. NR1-positive sequences from patients with anti-NMDARE have been reported as more likely to have a low mutation rate or no mutations (39). Our study showed that the circulating BCRs were hypermutated, and the somatic hypermutation rate was lower in the anti-LGI1E group. We suggest that these changes in framework regions might help the complementary determining regions recognize antigens; therefore, they might be antigen-driven characteristics.

Many questions remain. Here, we examined peripheral circulating blood cells; however, many lymphocyte populations involved in the pathogenesis of autoimmune encephalitis are present in the brain parenchyma and cerebrospinal fluid. This study included a small number of subjects, and more samples are needed for further exploration. The biological functions of common clones shared by patients with anti-NMDARE and anti-LGI1E need to be investigated.

## Data Availability Statement

The datasets presented in this study can be found in online repositories. The names of the repository/repositories and accession number(s) can be found below: https://ngdc.cncb.ac.cn/gsa-human/browse/HRA001102, HRA001102.

## Ethics Statement

The studies involving human participants were reviewed and approved by the Ethics Committee of the Institutional Review Board of Peking Union Medical College Hospital (PUMCH) (IRB JS-891). Written informed consent to participate in this study was provided by the participants’ legal guardian/next of kin.

## Author Contributions

The project was mainly conceived by JF. Sample preservation and preparation of antigen were conducted by YS. Flow cytometry and single-cell sequencing were conducted by YS and JF. Data were analyzed by JF. The manuscript was mainly drafted by JF. YS provided assistance to this work. The manuscript was revised by JF and SF. Samples and the clinical information of patients were collected by SF and HR. The research was supervised by HG and JW. All authors contributed to the article and approved the submitted version.

## Funding

This work was supported by the CAS Key Laboratory of Mental Health, Institute of Psychology, Chinese Academy of Sciences and Peking Union Medical College Hospital Youth Program (Grant No. pumch201911390).

## Conflict of Interest

The authors declare that the research was conducted in the absence of any commercial or financial relationships that could be construed as a potential conflict of interest.

## Publisher’s Note

All claims expressed in this article are solely those of the authors and do not necessarily represent those of their affiliated organizations, or those of the publisher, the editors and the reviewers. Any product that may be evaluated in this article, or claim that may be made by its manufacturer, is not guaranteed or endorsed by the publisher.
