# Supplementary material for: Comprehensive B-Cell Immune Repertoire Analysis of Anti-NMDAR Encephalitis and Anti-LGI1 Encephalitis
Source: Front Immunol. 2021 Oct 7;12:717598. doi: 10.3389/fimmu.2021.717598 (PMC8529218; doi:10.3389/fimmu.2021.717598)

**Supplementary Materials**

**Comprehensive B Cell immune repertoire analysis of Anti-NMDAR Encephalitis And Anti-LGI1 Encephalitis**

Jingjing Feng^1,2^*, Siyuan Fan^3^*, Yinwei Sun^1,2^, Haitao Ren^3^, Hongzhi Guan,^3^ Jing Wang^1,2^

1 Chinese Academy of Sciences Key Laboratory of Mental Health, Institute of Psychology, Beijing, China.

2 Department of Psychology, University of Chinese Academy of Sciences, Beijing, China.

3 Department of Neurology, Peking Union Medical College Hospital, Chinese Academy of Medical Sciences and Peking Union Medical College, Beijing, China.

*These authors have contributed equally to this work

Correspondence to: J. Wang, PhD

CAS Key Laboratory of Mental Health, Institute of Psychology, Chinese Academy of Sciences, 16 Lincui Road, Chaoyang District, Beijing 100101, China.

Department of Psychology, University of the Chinese Academy of Sciences, 19A Yuquan Rd, Shijingshan District, Beijing 100049, China.

E-mail: wangjing@psych.ac.cn

Correspondence also to: H.Z. Guan, MD

Department of Neurology, Peking Union Medical College Hospital, Chinese Academy of Medical Sciences and Peking Union Medical College, Shuaifuyuan, Wangfujing Street, Beijing 100730, China.

Email: guanhz@263.net

**This file includes:**

Supplementary Table 1. BCR heavy chain sequences of NR1-positive and LGI1-positive B cells from single cell sequencing.

Supplementary Table 2. BCR clones with significant difference among the anti-NMDARE, anti-LGI1E, and HC groups.

Supplementary Table 3. IGHV and IGHJ genes with significant differences among the anti-NMDARE, anti-LGI1E, and HC groups.

Supplementary Figure 1. Morisita index among samples from the anti-NMDARE, anti-LGI1E, and HC groups.

Supplementary Figure 2. Structure of B cell pool in each sample from the anti-NMDARE, anti-LGI1E, and HC groups.

**Supplementary Tables**

**Supplementary Table 1. BCR heavy chain sequences of NR1-positive and LGI1-positive B cells from single cell sequencing.**

| **NR1-positive cells** | | | **LGI1-positive B cells** | | |
| --- | --- | --- | --- | --- | --- |
| **IGHV** | **IGHJ** | **CDR3 amino acid** | **IGHV** | **IGHJ** | **CDR3 amino acid** |
| IGHV3-21 | IGHJ4 | CARDALGGSSWNPFDYW | IGHV3-7 | IGHJ4 | CAREFDYW |
| IGHV3-48 | IGHJ4 | CARGSSSGWYKQNYLEYW | IGHV3-53 | IGHJ2 | CAREMSYYDSSGYYRQTRNWYFDLW |
| IGHV3- | IGHJ4 | CARGGYPDYW | IGHV1-2 | IGHJ6 | CARGFVYSSSWYRLDYYYYGMDVW |
| IGHV1-46 | IGHJ6 | CAREDYPHYGMDVW | IGHV3-30 | IGHJ6 | CARDLFMGSGWGEYNYYYGMDVW |
| IGHV3-30-3 | IGHJ6 | CARDLFMGSGWGEYNYYYYGMDVW | IGHV3-48 | IGHJ6 | CARAPIFGVPIMLPHHYGMDVW |
| IGHV1-18 | IGHJ3 | CARGWYYSDSGTTPGAFDIW | IGHV3-48 | IGHJ6 | CARAPIFGVPIMLPHHYGMDVW |
| IGHV1-46 | IGHJ4 | CARDAVGATYADDW | IGHV4-59 | IGHJ4 | CARAKPATYYYGSGSRSPFDYW |
| IGHV4-61 | IGHJ5 | CARADCGFGCPGLNWFDPW | IGHV4-59 | IGHJ4 | CARAKPTTYYYGSGSRSPFDYW |
| IGHV3-30,IGHV3-30-5 | IGHJ6 | CAKVCGGDYSLPRWAYYGMDVW | IGHV4-59 | IGHJ3 | CARATYYYDISSYYKYVFDIW |
| IGHV3-30,IGHV3-30-5 | IGHJ6 | CAKVCGGDCSLPRWAYYGMDVW | IGHV4-31 | IGHJ4 | CARRSRYYYGSGSTGAFDYW |
| IGHV3-7 | IGHJ4 | CATSSDAPGNHW | IGHV5-51 | IGHJ4 | CAIRAGYSYGYGWYYFDYW |
| IGHV3-7 | IGHJ4 | CATSSDAPGNHW | IGHV4-31 | IGHJ3 | CARDLGGQLERRGVFDVW |
| IGHV3-33 | IGHJ4 | CAREAECSGTSCYYDYW | IGHV3-20 | IGHJ3 | CARDHIRLLLNRDAFDIW |
| IGHV3-23,IGHV3-23D | IGHJ3 | CAKSPYDILTGYRTNWDAFDIW | IGHV3-21 | IGHJ4 | CARDGYYYGSGRAFNDYW |
| IGHV4-39 | IGHJ5 | CARETTTVTRRFDPW | IGHV4-59 | IGHJ2 | CARGLAYGSGSYYFDLW |
| IGHV4-59 | IGHJ3 | CARATYYYDISSYYKYVFDIW | IGHV3-21 | IGHJ4 | CARDALGGSSWYPFDYW |
| IGHV1-18 | IGHJ4 | CARDNGRSATTTFDSW | IGHV3-9 | IGHJ3 | CASRSGWYLEGAFDIW |
| IGHV4-59 | IGHJ4 | CARDPYGSGRHDYW | IGHV1-2 | IGHJ4 | CARVLGRGGSCYSIW |
| IGHV3-74 | IGHJ4 | CAKRKYCTSTTCWGLVDYW | IGHV3-74 | IGHJ3 | CARDYAIRRHAFDIW |
|  |  |  | IGHV4-59 | IGHJ5 | CARETTTVTRRFDPW |
|  |  |  | IGHV1-46 | IGHJ6 | CAREDYPHYGMDVW |
|  |  |  | IGHV3-30 | IGHJ4 | CAKWSSSWGPFDYW |
|  |  |  | IGHV3-30 | IGHJ4 | CAKWSSSWGPSDYW |
|  |  |  | IGHV3-23 | IGHJ4 | CAKGGYTYGMSDYW |
|  |  |  | IGHV3-30 | IGHJ3 | CAKDLPTVGAFDIW |
|  |  |  | IGHV3-15 | IGHJ4 | CTKDYDILTGFDYW |
|  |  |  | IGHV4-39 | IGHJ4 | CASLWQWLGDDYW |
|  |  |  | IGHV3-21 | IGHJ4 | CARSPSQSAFDYW |

CDR3: complementary determining region 3

**Supplementary Table 2. BCR clones with significant difference among the anti-NMDARE, anti-LGI1E, and HC groups.**

| CDR3 amino acid | P value (pairwise compare) | **Padj** | Note | Incidence in | | |
| --- | --- | --- | --- | --- | --- | --- |
|  |  |  |  | anti-NMDARE (N=9) | anti-LGI1E (N=10) | HC  (N=4) |
| CARHAKQLPFDPW | 0.013 | 0.039* | anti-NMDARE > anti-LGI1E | 4 | 0 | 0 |
|  | 0.058 | 0.173 | anti-NMDARE > HC |  |  |  |
| CAREEWFHMDIW | 0.013 | 0.039* | anti-NMDARE > anti-LGI1E | 4 | 0 | 0 |
|  | 0.058 | 0.173 | anti-NMDARE > HC |  |  |  |
| CARGGYSSSWYYFDYW | 0.002 | 0.006** | HC > anti-LGI1E | 1 | 0 | 3 |
|  | 0.012 | 0.035* | HC > anti-NMDARE |  |  |  |
| CARGVAARPARLGMDVW | 0.013 | 0.039* | anti-NMDARE > anti-LGI1E | 4 | 0 | 0 |
|  | 0.058 | 0.173 | anti-NMDARE > HC |  |  |  |

CDR3: complementary determining region 3; anti-NMDARE: anti-N-methyl-D-aspartate receptor; anti-LGI1E: anti-leucine-rich glioma-inactivated 1; HC: healthy control; *Padj<0.05, **Padj<0.01.

**Supplementary Table 3. IGHV and IGHJ genes with significant differences among anti-NMDARE, anti-LGI1E, and HC groups.**

| Gene name | P value | P value (pairwise compare) | **Padj** | Note |
| --- | --- | --- | --- | --- |
| IGHV1-67 | 0.029* | 0.014 | 0.043* | anti-NMDARE > anti-LGI1E |
| IGHV3-71 | 0.029* | 0.012 | 0.036* | HC > anti-NMDARE |
| IGHV4-4 | 0.008* | 0.002 | 0.006** | anti-NMDARE > anti-LGI1E |
| IGHJ4 | 0.024* | 0.009 | 0.028* | anti-NMDARE > HC |

Anti-NMDARE: anti-N-methyl-D-aspartate receptor; anti-LGI1E: anti-leucine-rich glioma-inactivated 1; HC: healthy control; *Padj<0.05, **Padj<0.01.

**Supplementary Figures**

**Supplementary Figure 1. Morisita index among samples from the anti-NMDARE, anti-LGI1E, and HC groups.**


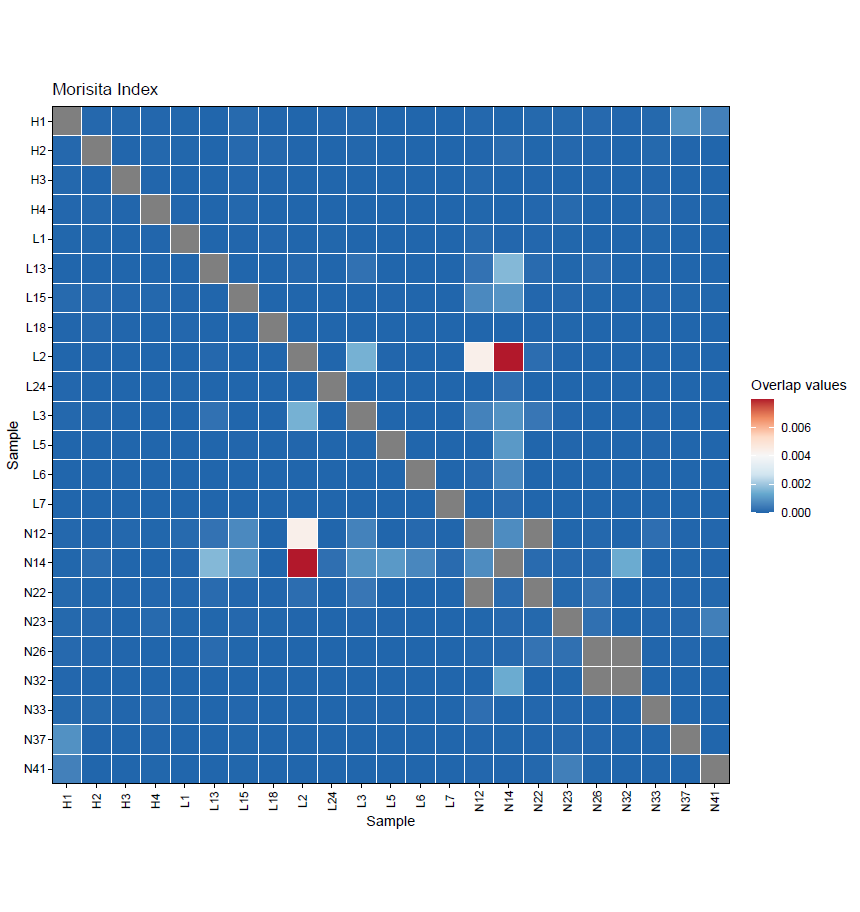


**Supplementary Figure 2. The clonality of each sample from the anti-NMDARE, anti-LGI1E, and HC groups.**


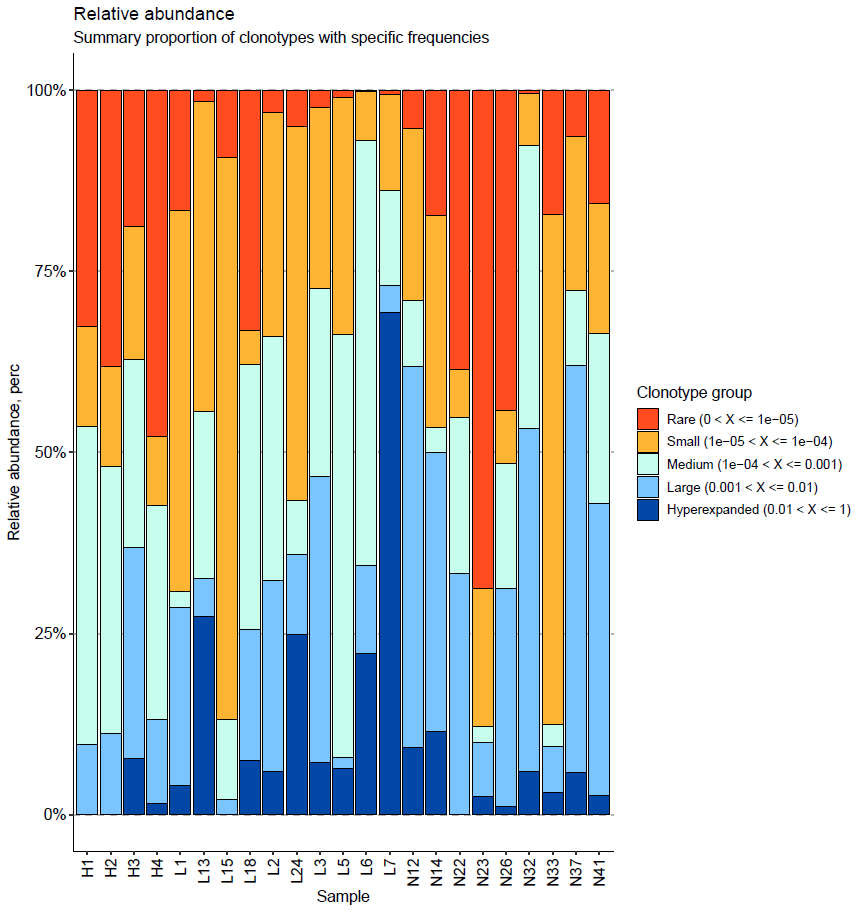

Supplement: Supplementary file 1 [file DataSheet_1.docx]
